# Employing transfer learning for breast cancer detection using deep learning models

**DOI:** 10.1371/journal.pdig.0000907

**Published:** 2025-06-16

**Authors:** Frimpong Twum, Charlyne Carol Eyram Ahiable, Stephen Opoku Oppong, Linda Banning, Kwabena Owusu-Agyemang

**Affiliations:** Department of Computer Science, Kwame Nkrumah University of Science and Technology, Kumasi, Ghana; University of Illinois Urbana-Champaign, UNITED STATES OF AMERICA

## Abstract

Breast cancer remains a critical global health concern, affecting countless lives worldwide. Early and accurate detection plays a vital role in improving patient outcomes. The challenge lies with the limitations of traditional diagnostic methods in terms of accuracy. This study proposes a novel model based on the four pretrained deep learning models, Mobilenetv2, Inceptionv3, ResNet50, and VGG16, which were also used as feature extractors and fed on multiple supervised learning models using the BUSI dataset. Mobiletnetv2, inceptionv3, ResNet50 and VGG16 achieved an accuracy of 85.6%, 90.8%, 89.7% and 88.06%, respectively, with Logistic Regression and Light Gradient Boosting Machine being the best performing classifiers. Using transfer learning, the top layers of the model were frozen, and additional layers were added. A GlobalAveragePooling2D layer was employed to reduce spatial dimensions of the input image. After training and testing based on the accuracy, ResNet50 performed the best with 95.5%, followed by Inceptionv3 92.5%, VGG16 86.5% and lastly Mobilenetv2 84%.

## Author summary

Breast cancer is one of the leading causes of mortality among women worldwide, and early detection plays a crucial role in improving survival rates. In this study, we explore the use of transfer learning, a technique that leverages pretrained deep learning models, to improve breast cancer classification using ultrasound images. We compare four well-established models—MobileNetV2, InceptionV3, ResNet50, and VGG16—evaluating their effectiveness in both feature extraction and fine-tuning approaches. Our findings show that ResNet50 and InceptionV3 outperform other models in accuracy, specificity, and robustness, making them strong candidates for clinical applications. MobileNetV2, while slightly less accurate, offers computational efficiency, making it suitable for real-time or resource-limited settings. By providing a comprehensive comparison of these models, our study contributes to the development of AI-driven breast cancer detection tools that can aid in faster, more reliable diagnoses.

## 1. Introduction

Breast cancer is the most prevalent cancer among women across the globe according to the World Health Organization (WHO). In 2022, 2.3 million cases and 670 000 deaths have been reported globally [1]. Breast cancer, a complex disease, primarily affecting women is a significant global health challenge. Prognostic factors in breast cancer, such as tumor size, lymph node involvement, hormone receptor status, and genetic markers, help predict the likely course of the disease and guide treatment decisions [2]. Although common methods such as clinical examination, mammography and biopsy [3,4] have immensely helped in breast cancer detection, the need for a more robust method for detecting breast cancer earlier cannot be overlooked [5].

In the medical field, artificial intelligence (AI), particularly machine learning (ML) has made significant strides, presenting an opportunity to offer solutions to the problems associated with detecting breast cancer [4,6,7]. Machine learning techniques can be used for the analysis of large/enormous volumes of medical images by leveraging on advanced computational algorithms to identify meaningful patterns and features, facilitating accurate diagnosis [8]. With regards to breast cancer detection, machine learning algorithms can be trained to learn patterns on labeled datasets that indicate cancerous and non-cancerous lesions. In contributing to a more precise and judicious diagnosis that are well timed, these algorithms can then be used to classify new, unseen images and also assist in the decision-making processes of healthcare professionals [9,10]. Also, in order to strengthen the field of breast cancer detection, conducting a comparative study of classification of algorithms has become essential for identifying the most accurate and efficient models for early diagnosis [11].

The objective of this study is to develop and implement a machine learning-based model capable of effectively and accurately detecting and classifying malignant and benign lesions, from medical imaging data, with the goal of enhancing breast cancer detection.

The remainder of this study is organized as follows: Section 2 provides a comprehensive literature review, outlining key concepts relevant to the study. Section 3 details the research methodology, including feature extraction techniques, the proposed model architecture, and the performance evaluation metrics. Section 4 presents a thorough analysis and discussion of the experimental results. Finally, Section 5 concludes the study by summarizing key findings and outlining directions for future research.

## 2. Related works

Ali et al. [12] employed both a meta-learning ensemble technique and also transfer learning to classify benign and malignant lesion using the Breast Ultrasound Images Dataset (BUSI). The meta-learning ensemble technique was to optimize the model’s learning process and enhance its adaptability to new and unseen datasets. Using transfer learning, models such as InceptionV3, ResNet50, and DenseNet121 were employed to enhance feature extraction, while data augmentation techniques were applied to artificially expand the training dataset, increasing its size and variability for improved model performance. After experimentation, the meta-learning ensemble technique model performed the best across various evaluation metrics, including accuracy, precision, recall, and F1 score. This highlighted the model’s potential as a valuable tool for breast cancer diagnosis.

Zakareya et al. [13] proposed a novel deep learning model using techniques from both deep learning and granular computing to enhance breast cancer classification accuracy. The dataset used was ultrasound images and breast histopathology images sourced from Cairo University. The parameters used for training the model includes using the Adam optimizer, employing a learning rate of 0.0001 across 100 epochs and a batch size of 32. To prevent overfitting, a dropout rate of 50% was applied and an early stopping technique, setting it to halt at 100 epochs. The model achieved an accuracy of 93% on ultrasound images and 95% on breast histopathology images. The innovative techniques incorporated into the model, such as granular computing, shortcut connections, learnable activation functions, and an attention mechanism, contributed substantially its performance.

Rahman et al. [14] proposed a novel computational framework centered around a ResNet50 model. This pretrained network was fine-tuned to classify mammogram images into benign or malignant categories using the INbreast dataset. The results of this approach reveal an impressive classification accuracy of 93%, outperforming other models trained on the same dataset. To further enhance the field of breast cancer diagnosis, future developments may involve exploring alternative deep convolutional networks, such as VGG and AlexNet architectures. Moreover, the continued refinement of DCNN parameters and adaptations for specific clinical scenarios could bolster the model’s accuracy and applicability.

Jafari and Karami [15] introduced a novel method based on feature extraction and reduction for the detection of breast cancer in mammography images. The study evaluated the proposed method using four different machine learning algorithms: neural network (NN), k-nearest neighbor (kNN), random forest (RF), and support vector machine (SVM). Notably, the NN-based classifier achieved an impressive accuracy of 92% on the RSNA dataset. Comparing their method with state-of-the-art approaches, the authors demonstrated its superiority, particularly in terms of accuracy and sensitivity. On the MIAS dataset, their approach attained an accuracy as high as 94.5%, and on the DDSM dataset, it reached an accuracy of 96%. These results underscore the effectiveness of their method in accurately diagnosing breast lesions and outperforming existing techniques. However, further research is needed to investigate potential limitations, computational considerations, and the integration of additional clinical and genomic data to enhance the system’s predictive capabilities.

Yadav et al. [16] employed a range of machine learning algorithms, deep learning algorithms, and hybrid approaches on diverse datasets. The algorithms, including SVM (Support Vector Machine), KNN (k-Nearest Neighbors), RF (Random Forest), DT (Decision Tree), NB (Naive Bayes), LR (Logistic Regression), ELM (Extreme Learning Machine), and deep learning algorithms. One main finding indicated the importance of feature extraction in determining the accuracy of the chosen prediction method. The study utilized different feature extraction techniques, such as Principal Component Analysis (PCA) and autoencoders. SVM outperformed the other classifiers under consideration. This finding underscores the importance of selecting the appropriate classification method in the pursuit of accurate diagnosis. The study also underscored the need for dimension reduction techniques to optimize dataset dimensions and suggested that ensemble ML techniques may hold promise in enhancing the performance of individual models.

Malathi [17] used three different deep learning models - ResNet50, InceptionV3, and Xception to identify defects in breast thermographic images. Various optimization techniques and learning rates were employed to enhance the accuracy and performance of these models. After comparing the three models, it was revealed each architecture possesses distinct strengths and weaknesses, and that parameter adjustments significantly impact model accuracy and performance. These findings indicate the application of these models in fields demanding high accuracy.

Hossain et al. [18] introduced a breast cancer classification method anchored in a transfer learning approach, leveraging the VGG16 model. Breast cancer, characterized in ultrasound images by irregular shapes, intensity variations, and increased vascularity, presents a distinct visual signature compared to benign conditions; however, effectively leveraging these images for accurate classification remains a significant challenge due to variability in image quality and tumor presentation. The inherent complexities stem from the presence of speckle noise and intricate textures within breast tissue. To mitigate the impact of speckle noise, a median filter was judiciously employed to enhance image quality. The pretrained VGG16 model’s convolutional layers, coupled with maxpooling layers, was harnessed as a feature extractor, while a novel two-layer deep neural network served as the classifier. Model optimization was achieved through the utilization of the Adam optimizer with a learning rate set at 0.001, while binary cross-entropy served as the loss function. To thwart overfitting, dropout layers within the hidden layers were strategically implemented. The authors consolidated breast ultrasound images from two distinct databases, amassing a total of 897 images. This amalgamation facilitated comprehensive training, validation, and testing of the classifier, gauging its performance and generalization capabilities. The model achieved a training accuracy of 98.2% and testing accuracy of 91%. To visualize and assess the model’s localization of targeted regions, the authors employed the Gradient Class Activation Mapping (Grad-CAM) technique, yielding noteworthy insights. These visualizations underscore the model’s aptitude for localizing critical regions during the final convolutional layer.

Han & Yin [19] embarked on a study with a two-fold approach: feature engineering and the development of a classification model. The authors begun by recognizing the importance of data pre-processing, a necessary step before diving into data analysis. A meticulous correlation-based feature selection method was employed to cherry-pick 16 features deemed most relevant. The core innovation layed in the creation of a hybrid algorithm that marries the concepts of meta-learning and Artificial Neural Networks (ANN). The hybrid algorithm presented in this paper represents a fusion of multiple meta-learning models, with their collective output serving as input features for the creation of ANN models. The model achieved 98.74% accuracy and an impressive 98.02% F1-score for breast cancer prediction. While the authors have made notable strides, there is always room for refinement and expansion. The feature engineering component of the framework could benefit from optimization, with the potential for improved accuracy through the use of ensemble methods for feature selection. This, in turn, would provide more robust input for subsequent classification algorithms.

Aljuaid et al. [20] proposed an innovative computer-aided diagnosis approach for breast cancer classification, harnessing the power of deep neural networks, transfer learning and data augmentation techniques. Their research revolved around publicly available breast cancer images from the BrakeHis dataset, comprising 7,909 images stemming from diagnoses of 82 diverse patients. Three deep neural networks (DNNs) were deployed for breast cancer image classification, underpinned by an imaging-based methodology. The dataset was divided into traning and testing sets using a 65:35 ratio. Using ResNet as the primary classifier, the model achieved remarkable average accuracies ranging from 97.81% to 99.70% in both binary and multi-class classification tasks. Future research opportunities may focus on further refining and optimizing the proposed methodology.

Joshi & Gaud [21] a performed a comparative analysis of two deep learning methods: MobileNetV2 and InceptionV3. InceptionV3 outperformed MobileNetV2 in terms of accuracy with 83.84% as compared to 82.54% but had a high computational complexity. This is due to the fact that it used a higher number of parameters. This was to shed light on their advantages and disadvantages in the domain of transfer learning-based breast cancer detection using ultrasound images.

Mahoro & Akhloufi [22] provided a comprehensive overview of recent research integrating deep learning into medical imaging for breast cancer diagnosis. The study stated that, to bolster accuracy, deep learning innovations, such as Transformers, have shown great promise. These techniques have demonstrated remarkable results in image classification, object detection, and segmentation. For instance, the Vision Transformer (ViT) model can classify breast images as malignant or benign, while the Conditional DEtection Transformer(detr)-ResNet50 model, with a ResNet-50 backbone, aided in breast cancer detection. Furthermore, segmentation methods like Transformer for Semantic Segmentation (TrSeg), can extract regions of interest for breast cancer diagnosis.

Behar & Shrivastava [23] proposed an effective CNN-based model, hinging on the pre-configured ResNet50 architecture, that exploits the transfer learning technique for automatic image classification between malignant and benign tumors, using histopathology images from the BreakHis dataset. Impressively, their endeavors culminate in a model that showcases state-of-the-art performance, boasting remarkable training, validation, and test accuracies of 99.70%, 99.24%, and 99.24%, respectively. These outcomes not only exceed previous studies but also affirm the model’s proficiency, with an accuracy increase of 0.66% in training and 0.2% in test results. Beyond accuracy, the authors highlighted noteworthy enhancements in average precision, F1 score, and the achievement of an impressive receiver operating characteristic (RoC) area of 99.1%. Moreover, the authors grappled with the complexities stemming from highly unbalanced and smaller sample images, an issue they tactfully address through data augmentation and judicious assignment of class weights.

Alanazi [24] set out to harness the potential of Convolutional Neural Networks (CNNs) in an effort to automate the identification of breast cancer. Their focus was on scrutinizing the hostile ductal carcinoma tissue zones within whole-slide images (WSIs). This approach aimed to enhance the accuracy of breast cancer detection and reduce diagnostic errors. The research used a dataset, having approximately 275,000 RGB image patches, each measuring 50 × 50 pixels. The automated system for breast cancer detection developed achieved an 87% accuracy rate. Crucially, the proposed system surpassed the performance of machine learning (ML) algorithms, yielding an 8% improvement in accuracy over the traditional algorithmic approach.

Kumar et al. [25] proposed using MobileNetV2 to classify tumors of late-stage breast cancer diagnoses in India. MobileNetV2 was chosen due to operational efficiency, characterized by fewer computational operations and also an ideal candidate for deployment on mobile devices, enhancing its accessibility and utility. The authors reported an overall model accuracy of 89%, a sensitivity rate of 89.92%, specificity rate of 86.84%, and an area under the receiver operating characteristic curve (AUC) score of 88.38%, confirming the model’s overall performance excellence. In spite of the gains using this model, the model can be validated across diverse datasets to assess the model’s generalizability and its ability to adapt to different population groups and clinical settings.

Naji et al. [26] applied five prominent machine learning algorithms: Support Vector Machine (SVM), Random Forests, Logistic Regression, Decision Tree, and KNN for breast cancer diagnosis using the Wisconsin Breast Cancer Diagnostic dataset (WBCD). These algorithms were evaluated based on various performance metrics, including accuracy, sensitivity, precision, and AUC. After a thorough comparison of the models, the study found that the Support Vector Machine had the highest accuracy rate of 97.2%, precision of 97.5%, and an AUC of 96.6%.

Bhise et al. [27] utilized a Convolutional Neural Networks (CNN) as a classifier model, coupled with Recursive Feature Elimination (RFE) for feature selection on the BreaKHis 400X Dataset. Five distinct algorithms—Support Vector Machine (SVM), Random Forest, k-Nearest Neighbors (KNN), Logistic Regression, and Naïve Bayes were also employed for a comparative analysis. For the CNN model, the Rectified Linear Unit (ReLU) activation function was used to predict outcomes in terms of probabilities. The CNN model was superior in terms of accuracy as well as precision when compared to the other supervised learning algorithms

Albashish et al. [28] addressed the challenge of computational cost and parameter requirements associated with Convolutional Neural Networks (CNNs). The study leveraged on the Visual Geometry Group with 16-layer deep architecture (VGG16) to extract high-level features from the BreaKHis benchmark histopathological image dataset. Subsequently, multiple machine learning classifiers were employed to handle various breast cancer histopathological image classification tasks, including binary and multiclass (eight-class) classifications. The experimental results on the public BreakHis benchmark dataset reveal the superior performance of the proposed model compared to previous works on the same dataset. The authors observed that the proposed model, in conjunction with Support Vector Machine (SVM) classifiers, excels in both binary and multiclass classification tasks, demonstrating remarkable improvements in specificity, particularly in multiclass classification. This suggests that the features extracted from VGG16 can discern complex breast cancer cases, thereby enhancing the diagnostic process. Future research endeavors should focus on investigating ensembles of different classifiers and pre-trained models to further elevate performance in this intricate domain of breast cancer diagnosis.

Ansar et al. [29] proposed an innovative approach for early breast cancer detection using a MobileNet-based architecture. The study achieved notable results, with accuracy rates of 86.8% for the Digital Database for Screening Mammography (DDSM) dataset and 74.5% for the Curated Breast Imaging Subset of DDSM (CBIS-DDSM). These results were superior to the performance of other deep convolutional neural network (CNN) models, including AlexNet, VGG16, GoogleNet, and ResNet. While the MobileNetV1 architecture delivered impressive performance, it is worth noting that MobileNetV2 offers a slight decrease in results while significantly reducing the number of parameters. The paper suggests potential improvements through data augmentation techniques and increasing the dataset size. To address these gaps and contribute to the field of breast cancer detection, there is a need to develop new methodologies that enhance the accuracy and efficiency of mass classification within mammograms. Future research could focus on optimizing MobileNet-based architectures further or exploring ensemble models to improve overall performance.

### 2.1 Comparisons with existing literature

This study builds upon and extends prior research in transfer learning for breast cancer detection by comparing multiple pretrained models and evaluating their effectiveness with various supervised learning classifiers. Unlike previous studies that focused on a single model or classifier, this work integrates both feature extraction and fine-tuned transfer learning approaches to provide a more comprehensive analysis.

In contrast to Ali et al. [12], which applied InceptionV3, ResNet50, and DenseNet121 using a meta-learning ensemble technique, this study examines four different pretrained models—MobileNetV2, InceptionV3, ResNet50, and VGG16—while also evaluating multiple supervised classifiers. By doing so, this study identifies the best-performing model-classifier combinations, offering a more adaptable and practical framework for real-world breast cancer detection. Zakareya et al. [13] proposed a hybrid deep learning and granular computing approach to improve classification accuracy. While their method relied on advanced optimization techniques, our study demonstrates that traditional deep learning architectures, when paired with effective classifiers like LightGBM and XGBoost, can achieve competitive or even superior results without the added computational complexity. The study by Rahman et al. [14] showcased the strong performance of ResNet50 for mammogram classification, achieving high accuracy. While their work focused on a single architecture, our study extends this analysis by comparing multiple models and demonstrating that ResNet50, alongside InceptionV3, consistently outperforms other models in feature extraction and fine-tuning tasks. This further substantiates ResNet50’s reliability for breast cancer classification, particularly when paired with high-performing classifiers. Hossain et al. [18] and Joshi & Gaud [21] explored individual model performances, such as VGG16 and MobileNetV2, respectively. While they validated the effectiveness of these models, they did not perform comparative evaluations against other architectures or integrate various machine learning classifiers. Our study not only aims to verify the utility of these models but also places them within a broader comparative framework, showing that models like ResNet50 and InceptionV3 provide better overall performance. Unlike Behar & Shrivastava [23], which focused solely on CNN-based classification using ResNet50, this study incorporates supervised learning classifiers to assess how different classifiers interact with deep learning-generated features. This hybrid approach offers a more nuanced understanding of breast cancer classification by demonstrating that combining deep learning and ensemble machine learning techniques leads to superior diagnostic accuracy.

This study is guided by the following research hypotheses. First, it is hypothesized that the application of transfer learning using pretrained models such as InceptionV3, ResNet50, and DenseNet121 significantly enhances the accuracy of breast cancer classification in ultrasound images. Second, the study posits that the classification performance varies considerably depending on the specific combination of pretrained model architecture and classifier employed. Finally, it is hypothesized that a systematic and optimized integration of model architecture with classifier selection will yield superior diagnostic performance compared to relying on either element independently. These hypotheses aim to extend current knowledge by offering a comprehensive analysis of how architectural and algorithmic choices interact to impact classification outcomes in medical imaging.

## 3. Methodology

**Fig 1:**
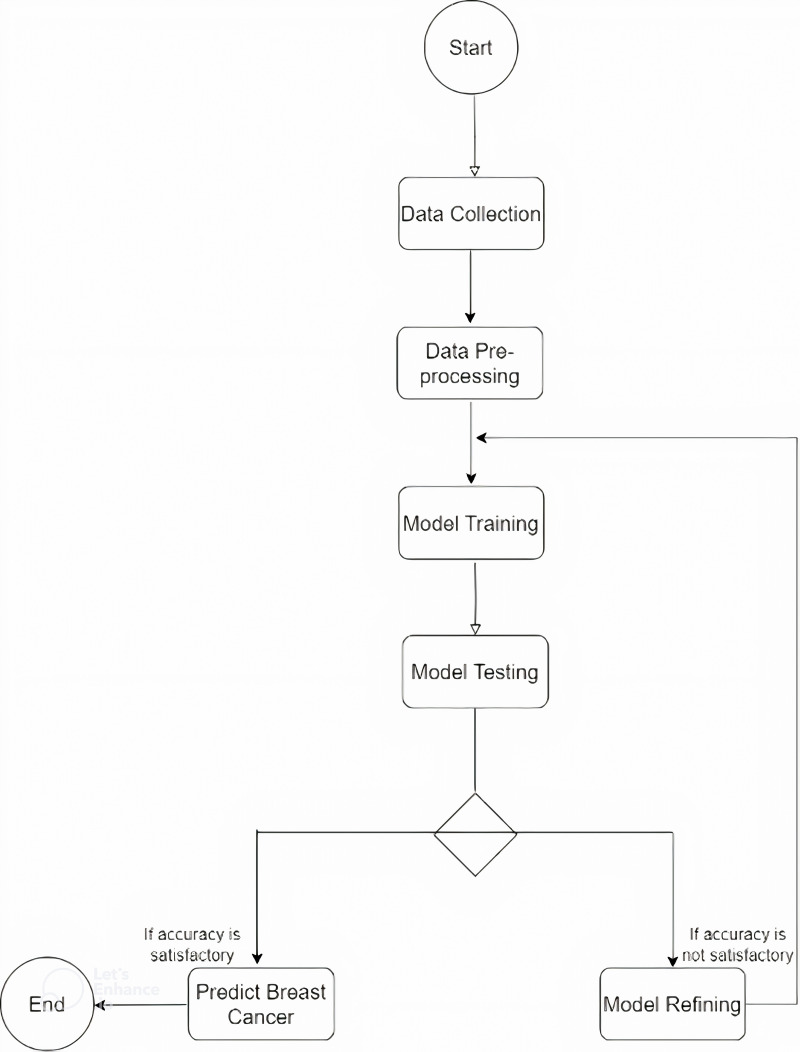
Flowchart diagram of model development.

This section outlines the steps employed in the breast cancer detection model as shown in Fig 1 based on four pretrained models: MobileNetV2, InceptionV3, ResNet50, and VGG16. These pretrained models were trained on the Breast Ultrasound Images (BUSI) dataset obtained from Kaggle [30] both as feature extractors and also as training models.

The BUSI dataset [30] comprises a comprehensive collection of ultrasound images, each accompanied by ground-truth annotations indicating the presence of either benign or malignant breast tumors. The dataset was collected in 2018 and includes data from 600 female patients, aged between 25 and 75 years. It contains a total of 1,312 images, with an average resolution of 500 × 500 pixels, all stored in PNG format. Each original ultrasound image is paired with a corresponding ground-truth mask, facilitating accurate lesion localization and classification. The dataset is categorized into two primary classes: benign and malignant, thereby providing a valuable resource for the development and evaluation of breast cancer classification models.

The dataset was pre-processed to ensure consistency and address any artifacts or noise present in the images and also split into Train, Test and Validation sets. The ratio used was 80:10:10 respectively. The primary preprocessing steps includes: Noise reduction using gaussian filtering [31], contrast enhancement using Adaptive histogram equalization to enhance contrast [32], making tumor regions more distinguishable from surrounding tissues and data augmentation, to increase dataset variability and prevent overfitting [33]. Data augmentation techniques implemented, included random rotations, horizontal and vertical flipping, scaling, and elastic deformations. These preprocessing steps ensured that the dataset maintained high-quality representations, improving the robustness and generalizability of the trained models.

Each of the four pretrained models - MobileNetV2, InceptionV3, ResNet50, and VGG16 was trained on the pre-processed BUSI dataset.

The pretrained models used are describe below:

- MobileNetV2 is designed to be lightweight and efficient, making it particularly useful for applications on mobile devices with limited computational resources. It uses depth wise separable convolutions, which split the standard convolution into two separate layers: a depth wise convolution and a pointwise convolution. This reduces the number of parameters and computations, leading to faster and more memory-efficient inference. MobileNetV2 is often used for tasks where real-time performance is important and computational efficiency is a priority [34].
- InceptionV3, also known as GoogLeNetv3, is known for its use of “inception modules,” which consist of multiple parallel convolutional layers with different filter sizes. These modules allow the model to capture features at different scales and resolutions. InceptionV3’s architecture enables it to efficiently learn complex patterns in images, making it well-suited for tasks that require a high level of image understanding. It is deeper and more complex than MobileNetV2, making it suitable for tasks where accuracy and understanding fine-grained details are important [35].
- VGG16 (Visual Geometry Group 16) is a well-known and relatively simple architecture that consists of 16 layers, including 13 convolutional layers and 3 fully connected layers. It is known for its uniform architecture, using small convolutional filters (3x3) with a small stride throughout the network. This results in deeper feature extraction. VGG16 has been influential in the development of deeper CNNs and is often used as a baseline for image classification tasks. However, its large number of parameters can make it computationally expensive [36].
- ResNet50 (Residual Network 50) is part of the ResNet family of architectures and is known for its deep structure. It addresses the problem of vanishing gradients in very deep networks using residual connections. Residual connections allow the network to skip certain layers and propagate gradients more effectively, enabling the training of very deep networks. ResNet50 is highly effective in learning intricate features from images and has achieved state-of-the-art performance on various image classification benchmarks [37].

The model was evaluated using these metrics derived from the confusion matrix [38] and they are described below:

• **Accuracy:** Accuracy gives an estimate of how well the model is likely to perform on new, unseen data. It helps assess the model’s generalization ability and its ability to make accurate predictions on data it hasn’t encountered during training. Accuracy is one of the most straightforward metrics and represents the ratio of correctly predicted instances to the total number of instances in the dataset.


$$\begin{array}{c} \text{Accuracy }=\frac{\text{Number\textbackslash{} of\textbackslash{} Correct\textbackslash{} Predictions}}{\text{Total\textbackslash{} Number\textbackslash{} of\textbackslash{} Predictions}} \end{array}$$ (1)

• **Precision:** Precision, also known as positive predictive value, measures the ratio of correctly predicted positive instances to the total number of instances predicted as positive. Precision focuses on minimizing false positives, making it important in cases where the cost of false positives is high. For example, in medical diagnoses, a high precision indicates a low rate of false positives, meaning that when the model predicts a positive case, it’s likely to be correct.


$$\begin{array}{c} \text{Precision }=\frac{\text{True\textbackslash{} Positives}}{\text{True\textbackslash{} Positives\textbackslash{} + \textbackslash{} False\textbackslash{} Positives}} \end{array}$$ (2)

• **Recall (sensitivity or true positive rate):** Recall measures the ratio of correctly predicted positive instances to the total number of actual positive instances in the dataset. Recall is particularly important when the goal is to minimize false negatives. In medical contexts like cancer detection, false negatives can be more detrimental, as they represent missed cases that require medical attention.


$$\begin{array}{c} \text{Recall }=\frac{\text{True\textbackslash{} Positives}}{\text{True\textbackslash{} Positives\textbackslash{} + \textbackslash{} False\textbackslash{} Negatives}} \end{array}$$ (3)

• **F1 score:** The F1 score is the harmonic mean of precision and recall, giving equal importance to both metrics. It balances precision and recall and provides a single metric that summarizes a model’s ability to make accurate positive predictions while minimizing false negatives. The F1 score ranges from 0 to 1, where 1 indicates perfect precision and recall, and 0 indicates poor performance. A higher F1 score is desirable, indicating a good trade-off between precision and recall.


$$\begin{array}{c} \text{F1\textbackslash{} Score }=2*\frac{\text{Precision\textbackslash{} *\textbackslash{} Recall}}{\text{Precision\textbackslash{} + \textbackslash{} Recall}} \end{array}$$ (4)

A thorough cross-validation process was performed to ensure unbiased evaluation and robustness.

### 3.1. Algorithm for breast cancer detection using pretrained models

a Load dataset BUSI (Breast Ultrasound Images) and corresponding labels
b Remove any missing or invalid data instances from the dataset.
c Normalize the image data to ensure consistent and standardized inputs.
d Split the dataset into training, testing and validation sets for model evaluation.
e Import the necessary libraries, including Tensorflow, Keras, and all other important packages.
f Load the pre-trained model using the appropriate keras function, tf.keras.applications.*pre-trained model*.
g Add a GlobalAveragePooling2D layer to reduce spatial dimensions.
h Integrate a dense layer prediction layer for Binary Classification.
i Compile the new model with an Adam optimizer using a base learning rate of 0.0001, a Binary Cross Entropy loss function suitable for binary classification, and monitoring of accuracy as a key metric during training.
j Initialize the model using the constructed architecture.
k Train the model using the pre-processed training dataset.
l Implement suitable training parameters, including initial epochs of 50 and fine-tune epochs of 50 (making 100 epochs in all) and batch size.
m If the model’s accuracy is not satisfactory, refine the model: Adjust hyperparameters (e.g., learning rate, dropout rate).
n Modify layers or architecture based on insights gained from testing.
o Reiterate model training using the updated configuration.
p Evaluate the trained model’s performance using the pre-processed testing dataset.
q Calculate accuracy, precision, recall, F1 score.

### 3.2. Algorithm for extracting features using pretrained models for breast cancer detection

a Load dataset BUSI (Breast Ultrasound Images) and corresponding labels
b Remove any missing or invalid data instances from the dataset.
c Normalize the image data to ensure consistent and standardized inputs.
d Split the dataset into training, testing and validation sets for model evaluation.
e Import the necessary libraries, including Tensorflow, Keras, and all other important packages.
f Load the pre-trained model using the appropriate keras function, tf.keras.applications.*pre-trained model*.
g Extract features form images using pretrained models
h Load features into supervised learning classifiers
i Evaluate the trained model’s performance using the pre-processed testing dataset.
j Calculate accuracy, precision, recall, F1 score.

## 4. Results and analysis

### 4.1 Model analysis using supervised learning architectures

Features were extracted from each of the pre-trained models at the bottleneck layer were fed into a variety of supervised learning classifiers to further provide more analysis on each of their performance in classifying breast cancer. The pre-trained models were used to extract meaningful features from the breast cancer dataset. The last layer before the fully connected layer for classification, commonly called the bottleneck layer [39], is used in this study. A description of the pre-trained network and the layers used are presented in Table 1.

**Table 1:** Pretrained networks.

Network	Image input size	Depth	Layer
Inceptionv3	299-by-299	48	avg_pool
MobileNetv2	224-by-224	53	global_average_pooling2d_1
ResNet50	224-by-224	50	avg_pool
VGG16	224-by-224	16	fc7

The extracted features were then used as input for several supervised classifiers. These classifiers are Light Gradient Boosting Machine (lightgbm), Extreme Gradient Boosting (xgboost), Logistic Regression (lr), Gradient Boosting Classifier (gbc), K Neighbors Classifier (knn), Extra Trees Classifier (et), Random Forest Classifier (rf), Ada Boost Classifier (ada), SVM - Linear Kernel (svm), Ridge Classifier (ridge), Decision Tree Classifier (dt), Linear Discriminant Analysis (lda), Quadratic Discriminant Analysis (qda) and Naive Bayes (nb) etc. Here are the results obtained for each of the pre-trained models’ feature extractions combined with different classifiers:

#### 4.1.1 MobilenetV2.

MobilenetV2, combined with various classifiers, demonstrated solid performance. Among the classifiers, LightGBM emerged as the best performer, achieving an accuracy of 85.6%, an AUC of 0.9031, and an F1-score of 0.7438. This indicates that MobilenetV2, when paired with LightGBM, effectively captures, and classifies the features related to breast cancer. Table 2 below illustrate the detailed accuracy and performance matric across the various classifiers, respectively.

**Table 2:** Performance metrics of classifiers with MobilenetV2.

Classifier	Accuracy	AUC	Recall	Precision	F1
AdaBoost (ada)	0.7998	0.8255	0.6284	0.7182	0.6661
Decision Tree (dt)	0.7217	0.6742	0.5428	0.5734	0.5545
Extra Trees (et)	0.8217	0.8916	0.543	0.8477	0.6586
Gradient Boosting (gbc)	0.8389	0.8854	0.6289	0.8323	0.7126
KNN	0.8246	0.852	0.6378	0.7799	0.6999
LDA	0.6454	0.6269	0.5522	0.462	0.4999
LightGBM	0.856	0.9031	0.6612	0.8657	0.7438
Logistic Regression (lr)	0.8389	0.8607	0.656	0.811	0.7202
Naive Bayes (nb)	0.5395	0.6276	0.8073	0.395	0.5295
QDA	0.6435	0.6019	0.4866	0.4508	0.4647
Random Forest (rf)	0.8198	0.8807	0.5101	0.8819	0.64
Ridge	0.7693	0.7705	0.605	0.6562	0.6249
SVM	0.7912	0.785	0.6047	0.7564	0.6319
XGBoost	0.8417	0.8936	0.6557	0.8231	0.7238

- High Performers: While LightGBM achieved the highest accuracy of 85.60% and the highest AUC of 0.9031, XGBoost followed closely with an accuracy of 84.17% and an AUC of 0.8936. These results suggest that both LightGBM and XGBoost effectively leverage the feature representations provided by MobilenetV2.
- Middle Performers: Gradient Boosting (GBC) and Logistic Regression (LR) also performed well, with accuracies above 83%. GBC’s high precision and F1-score indicate its balanced performance, making it a reliable choice for this task.
- Poor Performers: Naive Bayes (NB) and QDA were among the worst performers, with NB achieving only 53.95% accuracy. These models struggled with the feature space created by MobilenetV2, leading to lower overall performance.

#### 4.1.2 InceptionV3.

InceptionV3 stands out as the most effective model in this study, with Logistic Regression as its top-performing classifier. The combination achieved an accuracy of 90.84%, an AUC of 0.95, and an F1-score of 0.8482. These results suggest that InceptionV3 is highly capable of distinguishing between benign and malignant cases. Table 3 below show the performance matrices across the difference learning classifiers.

**Table 3:** Performance metrics of classifiers with InceptionV3.

Classifier	Accuracy	AUC	Recall	Precision	F1
AdaBoost (ada)	0.8599	0.9197	0.7091	0.8359	0.7653
Decision Tree (dt)	0.7807	0.7516	0.665	0.6611	0.6598
Extra Trees (et)	0.8713	0.9399	0.6377	0.9452	0.7596
Gradient Boosting (gbc)	0.8837	0.9368	0.721	0.8983	0.7972
KNN	0.8646	0.9242	0.7741	0.801	0.7859
LDA	0.7626	0.7503	0.6766	0.6265	0.6478
LightGBM	0.8951	0.9494	0.7566	0.9064	0.8212
Logistic Regression (lr)	0.9084	0.95	0.8098	0.9002	0.8482
Naive Bayes (nb)	0.6415	0.7396	0.8069	0.4786	0.5976
QDA	0.6826	0.6183	0.4389	0.5187	0.4721
Random Forest (rf)	0.8541	0.9336	0.5961	0.9202	0.7218
Ridge	0.8674	0.7724	0.7624	0.8158	0.7862
SVM	0.9028	0.8482	0.8282	0.8702	0.8437
XGBoost	0.8885	0.9445	0.7414	0.8986	0.8092

- Top Performers: Logistic Regression (LR) with an accuracy of 90.84% and AUC of 0.95 outperformed all other classifiers when using features from InceptionV3. LightGBM and XGBoost also performed strongly, with accuracies of 89.51% and 88.85%, respectively, and high AUC scores. These results indicate that the feature representations from InceptionV3 are particularly well-suited to these classifiers. LR achieves the highest accuracy and AUC, making it the most reliable classifier in terms of correctly identifying both positive and negative cases. Its high precision and recall balance result in a strong F1-score, indicating consistent performance across all metrics.
- Middle Performers: Gradient Boosting (GBC) and Extra Trees (ET) showed consistent performance, with GBC achieving an accuracy of 88.37% and ET with 87.13%. Both models maintained a good balance across all metrics, making them reliable options.
- Poor Performers: Like the results with MobilenetV2, Naive Bayes (NB) and QDA were the lowest-performing classifiers. QDA showed inferior performance with an accuracy of 68.26%, suggesting that it struggles with the high-dimensional feature space of InceptionV3.

#### 4.1.3 ResNet50.

ResNet50, renowned for its deep residual learning capabilities, addresses the vanishing gradient problem using skip connections, making it easier to train deep models while capturing more complex patterns in the data. When applied to breast cancer classification, ResNet50 demonstrated impressive performance across various classifiers, with Logistic Regression leading the pack. Logistic Regression paired with ResNet50 achieved an accuracy of 89.7%, an AUC of 0.9323, and an F1-score of 0.8288. This high performance indicates that ResNet50, combined with Logistic Regression, effectively captures, and classifies complex patterns in breast cancer data. Table 4 summarizes the performance metrics of the classifiers with ResNet50.

**Table 4:** Performance metrics of classifiers with ResNet50.

Classifier	Accuracy	AUC	Recall	Precision	F1
AdaBoost (ada)	0.8646	0.9074	0.7409	0.8229	0.775
Decision Tree (dt)	0.775	0.7446	0.6612	0.6529	0.654
Extra Trees (et)	0.8722	0.9335	0.6732	0.908	0.7689
Gradient Boosting (gbc)	0.8827	0.9377	0.7176	0.9019	0.7949
KNN	0.8646	0.913	0.7444	0.8209	0.7793
LDA	0.6091	0.6067	0.576	0.4203	0.4847
LightGBM	0.8846	0.9433	0.7295	0.9011	0.8006
Logistic Regression (lr)	0.897	0.9323	0.783	0.8904	0.8288
Naive Bayes (nb)	0.5901	0.6954	0.8636	0.4358	0.5776
QDA	0.6501	0.6018	0.4661	0.4709	0.4629
Random Forest (rf)	0.8703	0.9292	0.6494	0.9274	0.7602
Ridge	0.8637	0.7536	0.7536	0.8129	0.7799
SVM	0.8389	0.7513	0.7513	0.8011	0.7585
XGBoost	0.8856	0.9408	0.7325	0.9001	0.8039

- High performers: like Logistic Regression and XGBoost, with accuracies of 89.7% and 88.56% respectively, highlight the model’s ability to work effectively with classifiers that benefit from deep learning feature extraction.
- Middle performers: such as LightGBM and Gradient Boosting (GBC) also showed solid results, with accuracies above 88%, making them suitable alternatives depending on specific needs. However, poor performers like Naive Bayes and QDA, which achieved lower accuracies of 59.01% and 65.01% respectively, struggled with ResNet50’s complex feature space.
- ResNet50’s performance remains strong across various classifiers, indicating that the features it extracts are highly informative and generalizable. Although, the model’s depth and residual learning capabilities make it a strong contender, its computational demands may limit its use in resource-constrained environments.

#### 4.1.4 VGG16.

VGG16, while slightly behind InceptionV3 and ResNet50, still demonstrated an impressive performance with LightGBM as its top classifier. The combination achieved an accuracy of 88.08%, an AUC of 0.9304, and an F1-score of 0.7959. These results suggest that VGG16, while simpler than models like InceptionV3 and ResNet50, still effectively captures relevant features for classification. Table 5 details the performance metrics of classifiers used with VGG16.

**Table 5:** Performance metrics of classifiers with VGG16.

Classifier	Accuracy	AUC	Recall	Precision	F1
AdaBoost (ada)	0.8408	0.8875	0.7307	0.7683	0.7444
Decision Tree (dt)	0.7368	0.7064	0.6206	0.5891	0.6027
Extra Trees (et)	0.8665	0.9259	0.6889	0.8691	0.7665
Gradient Boosting (gbc)	0.8779	0.9204	0.7216	0.8788	0.7899
KNN	0.8542	0.8955	0.7479	0.791	0.7676
LDA	0.6015	0.5921	0.5731	0.4129	0.4783
LightGBM	0.8808	0.9304	0.7306	0.8794	0.7959
Logistic Regression (lr)	0.8284	0.8484	0.6857	0.7616	0.7174
Naive Bayes (nb)	0.5329	0.6178	0.7539	0.3835	0.5076
QDA	0.448	0.5914	0.985	0.3672	0.5348
Random Forest (rf)	0.8723	0.9185	0.6801	0.9004	0.7724
Ridge	0.7607	0.6146	0.6026	0.6411	0.6182
SVM	0.7246	0.5635	0.4645	0.7056	0.4996
XGBoost	0.8799	0.9285	0.7452	0.8637	0.7989

- High performers such as LightGBM and XGBoost, with accuracies of 88.08% and 87.99% respectively, demonstrate that these classifiers can effectively leverage the feature extraction capabilities of VGG16.
- Middle performers like Gradient Boosting (GBC) and Random Forest (RF) also showed robust performance, with accuracies above 87%, making them reliable choices.
- However, poor performers like QDA and Naive Bayes, with accuracies of 44.8% and 53.29% respectively, struggled to utilize VGG16’s feature space, leading to a higher rate of errors consistent with their results on other pre-trained models.

VGG16 combined with Gradient Boosting and LightGBM provided a good trade-off between sensitivity and specificity, making them reliable choices for breast cancer classification. Despite its simplicity, VGG16 remains an effective model, particularly when paired with strong classifiers like LightGBM, although its substantial number of parameters may lead to overfitting in some cases.

#### 4.1.5 Classifier performance across models.

Across all pre-trained models, LightGBM, XGBoost, and Logistic Regression consistently delivered the highest performance in terms of accuracy, AUC, and F1-score. These classifiers were able to effectively leverage the features extracted by the pre-trained models, resulting in high classification accuracy and balanced performance across all metrics.

**LightGBM:** LightGBM was the best-performing classifier for both MobilenetV2 and VGG16. Its ability to manage large datasets and its efficiency in finding the best split points make it a powerful tool for classification tasks. Its high accuracy and AUC scores across different models demonstrate its robustness and adaptability to various feature sets.

**Logistic regression:** Logistic Regression outperformed other classifiers for InceptionV3 and ResNet50. Its simplicity, interpretability, and robust performance on these models highlight its effectiveness in linear separability scenarios, making it a reliable choice for classification tasks.

In contrast, Naive Bayes and Quadratic Discriminant Analysis consistently underperformed, particularly in terms of accuracy and AUC. These models struggled to manage the complexity of the extracted features, leading to lower precision and F1-scores.

**Naive Bayes:** Naive Bayes consistently underperformed across MobilenetV2, InceptionV3, and ResNet50. This can be attributed to its strong assumptions about the independence of features, which may not hold true for complex datasets like those derived from deep learning models. Its low precision and F1-scores indicate that it frequently misclassifies samples, leading to high false positive rates.

**Quadratic discriminant analysis (QDA):** QDA was the worst-performing classifier for VGG16. Despite its high recall, which indicates a strong ability to identify positive cases, its extremely low precision leads to a large number of false positives, making it an unreliable classifier for this task.

InceptionV3 and ResNet50 provided the most robust feature extractions, leading to the best overall performance when combined with the top classifiers. MobilenetV2 and VGG16 also performed well, but with slightly lower accuracy and AUC values compared to InceptionV3 and ResNet50.

Based on the analysis, InceptionV3 emerges as the overall best model, particularly when paired with Logistic Regression. The combination of InceptionV3’s multi-scale feature extraction capabilities and Logistic Regression’s simplicity and effectiveness results in the highest accuracy, AUC, and F1-score across all models.

Despite the outstanding performance demonstrated by the best classifiers, it is equally important to discuss the performance of other classifiers that fall between these extremes. This will provide a more suitable for datasets where the signal-to-noise ratio is well balanced.

The middle-performing classifiers provide a broad range of options depending on the specific needs of the classification task. Ensemble methods like GBC, ET, and RF offer robustness and reliability, particularly in handling high-dimensional data. KNN, while simpler, can be effective with the right choice of parameters and a well-prepared dataset. AdaBoost is useful in situations where boosting weaker models is beneficial, though care must be taken to avoid overfitting.

These classifiers fill the gap between the best and worst performers, offering solid performance for scenarios where the top-performing models may be too resource-intensive or where a balance between complexity and accuracy is desired.

The results of this analysis underscore the superiority of LightGBM, XGBoost, and Logistic Regression when combined with pre-trained models for breast cancer classification. These classifiers demonstrated high accuracy, strong discriminative power (AUC), and balanced recall and precision, making them the preferred choice for this task.

The comparative analysis of MobilenetV2, InceptionV3, ResNet50, and VGG16 highlights the strengths and weaknesses of each model in the context of breast cancer classification. While all models performed well, InceptionV3 stands out as the most effective, especially when paired with Logistic Regression followed by ResNet50. This combination not only achieves the highest performance metrics but also provides a balance between model complexity and interpretability.

On the other hand, models like VGG16 and MobilenetV2 also show impressive performance, particularly when paired with LightGBM, making them suitable alternatives depending on the specific needs of the application, such as computational resources and model interpretability.

Classifiers like Naive Bayes and QDA, however, consistently underperform, indicating that they may not be suitable for the tasks.

**Fig 2:**
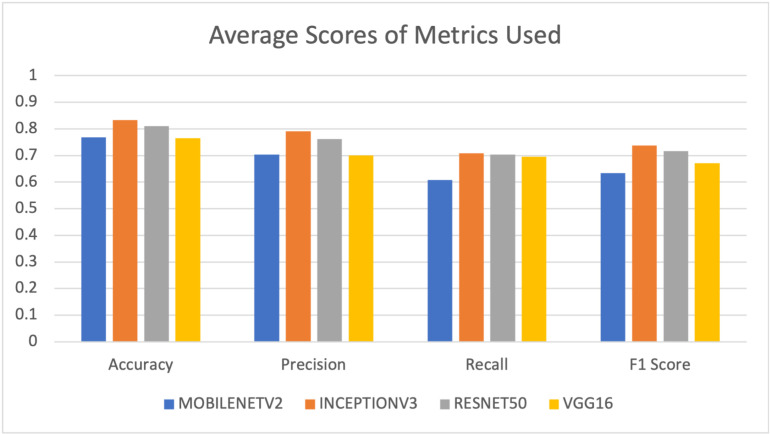
Average scores with pretrained model features.

Inceptionv3 consistently outperforms the other models across all metrics, with the highest average scores for accuracy, precision, recall, and F1 score as shown in Fig 2. It offers a well-balanced and robust performance. ResNet50 also demonstrates strong and balanced performance, particularly in terms of precision and recall, with the second-highest average scores. VGG16 and Mobilenetv2 perform competitively but with slightly lower average scores, indicating that they may be a bit less accurate and less robust overall.

From Table 6, using the pretrained models as feature extractors, VGG16 had the longest time (722.38s), making it the most computationally expensive model. MobileNetV2 was the fastest (112.93s), confirming its efficiency as a lightweight model whiles ResNet50 (212.25s) and InceptionV3 (347.34s) fall in between, with ResNet50 being more efficient than InceptionV3.This means that MobileNetV2 is best for speed, making it ideal for real-time applications or resource-limited environments.

**Table 6:** Time taken for feature extraction.

Model	Time (in sec)
inceptionv3	347.344629
mobilenetv2	112.932304
resnet50	212.249004
vgg16	722.38496

Computational efficiency is a crucial factor in deploying deep learning models for medical image classification, particularly in resource-constrained environments. MobileNetV2 emerges as a strong candidate for such settings due to its lightweight architecture and reduced computational requirements. Designed for efficiency, MobileNetV2 employs depthwise separable convolutions, significantly reducing the number of parameters and FLOPs (floating point operations per second) while maintaining competitive classification performance. This makes it an ideal choice for edge devices and real-time applications where computational resources are limited. Conversely, ResNet50 and VGG16, despite their superior classification performance, are computationally intensive due to their deeper architectures and large parameter counts. Optimizations for these models can be achieved through techniques such as model pruning, quantization, and knowledge distillation.

### 4.2 Model analysis using fine tuned pretrained models

The models were fine-tuned and optimized using transfer learning to suit the specific task of breast cancer detection. A GlobalAveragePooling2D layer was added to reduce spatial dimensions and a dense prediction layer for binary classification.

The trained models were evaluated using various performance metrics, such as accuracy, precision, recall, and F1 score.

**Fig 3:**
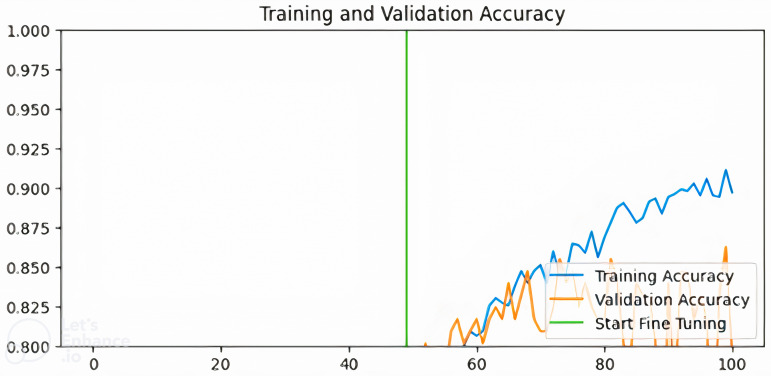
Training and validation accuracy for MobilenetV2-based model.

**Fig 4:**
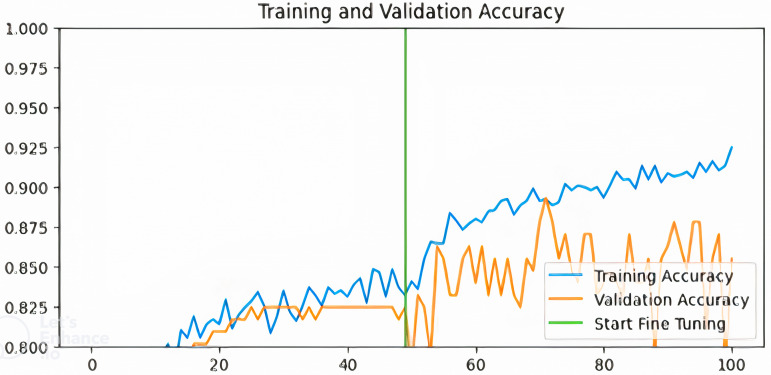
Training and validation accuracy for InceptionV3-based model.

**Fig 5:**
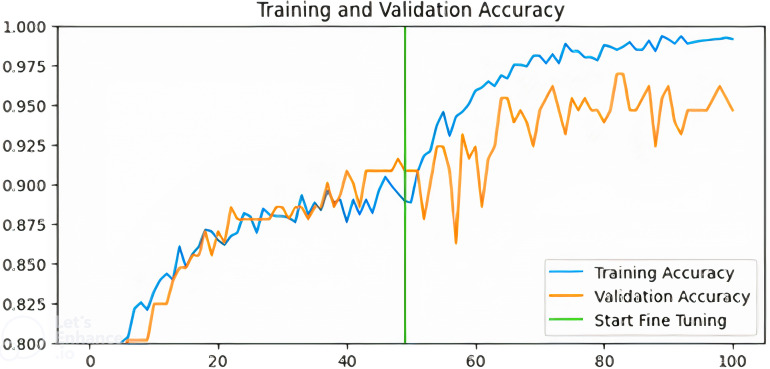
Training and validation accuracy for ResNet-based model.

**Fig 6:**
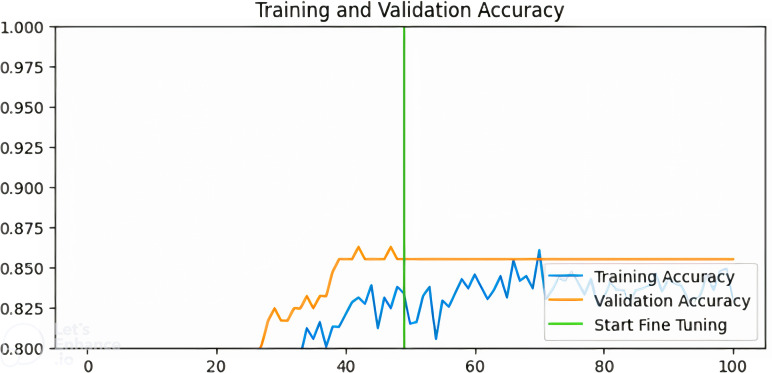
Training and validation accuracy for VGG-based model.

Figs 3–6 show the training and validation accuracies of the pre-trained models after fine-tuning for 50 epochs:

From Figs 3–6 above, the validation accuracy of the VGG16-based model plateaus early and does not show substantial improvement after fine-tuning, unlike ResNet50 and InceptionV3. This underperformance can be attributed to several architectural limitations. VGG16 follows a sequential deep learning architecture without shortcut connections, unlike ResNet50, which employs residual connections to mitigate the vanishing gradient problem. These residual connections help maintain stable gradient propagation during backpropagation, allowing deeper networks like ResNet50 to learn more complex patterns effectively.Additionally, VGG16 relies heavily on fully connected layers with a high number of parameters, increasing the risk of overfitting when fine-tuning on a relatively small dataset like BUSI.

In contrast, InceptionV3 incorporates factorized convolutions and multi-scale feature extraction, allowing it to adapt better to variations in medical images. Moreover, VGG16 lacks batch normalization layers, which are present in ResNet50 and InceptionV3. Batch normalization helps stabilize training, improve convergence speed, and reduce internal covariate shift. The absence of this feature in VGG16 likely contributes to its slower learning dynamics and early plateau during fine-tuning. These findings suggest that while VGG16 is a competent feature extractor, it may not be the optimal choice for fine-tuning in breast cancer classification compared to more modern architectures like ResNet50 and InceptionV3.

**Fig 7:**
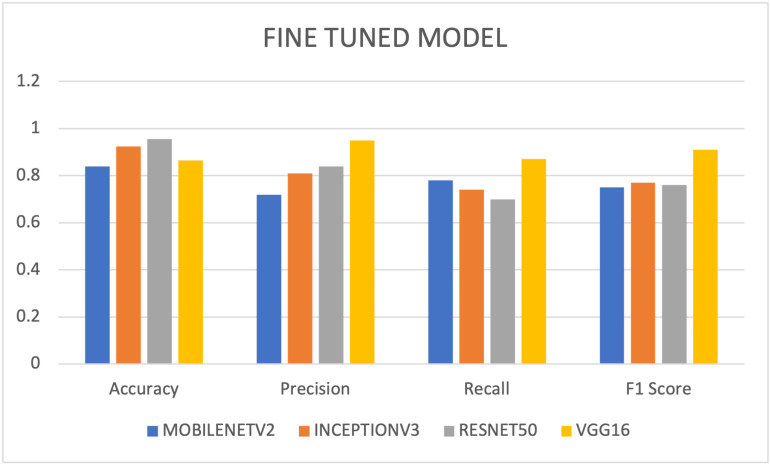
Performance metrics using fine-tuned model.

As shown in Fig 7, ResNet50 achieved the highest accuracy with 95.5%, indicating its overall performance in breast cancer detection on the BUSI dataset. VGG16 had an accuracy score of 87.5, precision score: 95%, recall score: 87%, and F1 score: 91%, making it a strong contender for breast cancer detection with a focus on correctly identifying positive cases. InceptionV3 demonstrated a balanced performance with competitive test accuracy of 92.5%, accuracy score of 68.75%, precision score of 84%, recall score of 70%, and F1 score of 76%. MobileNetv2 had a comparatively lower values, with a test accuracy of 84%, accuracy score of 62.5%, precision score of 72% but showed promising recall of 78% and F1 score of 75%. Based on these findings, ResNet50 stand out as the top-performing model for breast cancer detection on the BUSI dataset.

Four (4) pretrained deep learning models, namely MobileNetv2, InceptionV3, ResNet50, and VGG16 were evaluated and tested on BUSI dataset for breast cancer detection. The results after experimentation provided profound insights into their individual performances using transfer learning. With an accuracy of 95.5% after testing, ResNet50 emerged as the best performing model highlighting its effectiveness in breast cancer detection. VGG16 however demonstrated superior scores in terms of precision, recall, and F1 score, showing its prowess in accurately identifying positive breast cancer cases. InceptionV3 displayed a well-balanced performance, achieving competitive metrics in accuracy score, precision, recall, and F1 score. While MobileNetv2 obtained relatively lower accuracy scores and precision, it showed promising recall and F1 score. Based on the results, the ResNet50 holds promise for practical implementation in real-world medical practices, due to its robust overall performance. Data augmentation techniques and leveraging different layers during fine-tuning were implemented improving the performance of the models.

### 4.3 Discussion of results

The results of this study align with prior research indicating the efficacy of transfer learning in medical imaging. For example, studies by Armoogum et al. [40] and Alruily [41] have similarly demonstrated the superior performance of VGG16 in classifying breast cancer images, citing its ability to capture complex hierarchical features whiles Naas et al. [42] and Raza et al. [43] asserted same on ResNet models.

However, unlike these studies, our work extends the evaluation by incorporating multiple classifiers, providing a broader perspective on how model selection impacts classification outcomes. Additionally, our findings build upon Ali et al. [12], which compared different CNN architectures but did not incorporate various supervised learning classifiers. By integrating XGBoost and LightGBM in our approach, we offer additional insights into how hybrid models can enhance diagnostic precision. This hybridization is particularly relevant in cases where deep learning alone may be insufficient for complex decision-making, further reinforcing the need for explainable AI models in clinical environments.

By contextualizing these findings within existing literature and discussing their direct impact on clinical workflows, this study contributes to the ongoing conversation about AI-driven diagnostic solutions in breast cancer detection. As AI technologies continue to evolve, understanding their strengths, limitations, and practical usability in medical settings will be essential for ensuring their safe and effective integration into healthcare.

The findings of this study have several important implications for clinicians and medical practitioners seeking to integrate AI-assisted diagnostic tools into breast cancer detection workflows. One of the key takeaways is that ResNet50 and InceptionV3 demonstrated the highest classification accuracy and specificity, making them reliable choices for clinical applications where minimizing false positives and false negatives is critical. From a practical standpoint, the lower computational demands of MobileNetV2 make it a suitable option for deployment in resource-limited settings, such as remote healthcare centers or mobile diagnostic units. While MobileNetV2 exhibited slightly lower specificity, its efficiency in real-time processing could facilitate quicker preliminary screenings, which can then be followed by more in-depth analysis using high-performance models like ResNet50. Moreover, the confusion matrix analysis highlights the importance of selecting models with robust feature extraction capabilities to minimize misdiagnoses. False negatives, which were more prevalent in VGG16, pose a significant risk in clinical practice, as missing a malignant case could delay treatment and negatively impact patient outcomes. Therefore, while VGG16 remains a viable model, it should be supplemented with additional validation techniques, such as ensemble approaches or post-classification review by radiologists.

Despite the promising findings of this study, there are several limitations that warrant consideration. One key limitation is the diversity of the dataset. The BUSI dataset, while widely used in breast cancer detection studies, may not fully capture the variability present in real-world clinical settings. Differences in imaging equipment, patient demographics, and image quality could affect model generalizability. Additionally, there is a risk of overfitting, particularly when fine-tuning deep learning models on relatively small datasets. While transfer learning helps mitigate this issue by leveraging pretrained weights, further validation on larger, more diverse datasets is necessary to confirm the robustness of these models. Another important limitation pertains to the computational demands of certain models, such as ResNet50 and VGG16. While these architectures achieve high accuracy, their large number of parameters makes them computationally expensive for deployment in low-resource environments. Future research could explore optimization strategies, such as lightweight model architectures or cloud-based inference solutions, to improve deployment feasibility.

### 4.4 Advancing current knowledge

This study advances current knowledge in transfer learning for breast cancer detection by offering a systematic comparison of multiple pretrained deep learning models and evaluating their effectiveness across different classification techniques. Unlike previous studies that focus on single models or fine-tuning approaches in isolation, this research provides a dual-perspective analysis, examining both feature extraction and fine-tuning methodologies.

By integrating various supervised classifiers, such as LightGBM, XGBoost, and Logistic Regression, this study highlights the role of hybrid learning techniques in enhancing diagnostic accuracy. The comprehensive evaluation of multiple classifiers provides insights into how traditional machine learning techniques can complement deep learning-based feature extraction, leading to improved performance. The study also demonstrates the advantages of ensemble methods in enhancing classification reliability, particularly in scenarios where deep learning models alone may not provide sufficient generalizability.

Furthermore, the findings underscore the superior performance of ResNet50 and InceptionV3 in breast cancer classification, demonstrating their robustness when combined with ensemble learning methods. ResNet50, in particular, exhibited the highest accuracy, emphasizing its effectiveness in learning complex feature representations. The results also reveal that fine-tuning significantly boosts model performance compared to using pretrained models solely for feature extraction, reinforcing the importance of optimizing deep learning models for medical imaging tasks.

Additionally, this study contributes to the ongoing efforts to improve medical imaging diagnostics by establishing a structured framework for model evaluation. By testing different pretrained architectures on the BUSI dataset and systematically comparing their effectiveness, this work provides a benchmark for future research in transfer learning for breast cancer detection. The results indicate that selecting the right combination of model architecture and classifier can lead to more accurate and interpretable diagnostic systems, making AI-based detection tools more viable for clinical applications.

## 5. Conclusion and future works

Early diagnosis of breast cancer is vital in medical diagnostics since it may enhance patient outcomes. This study first extracted features form the BUSI dataset using four pre-trained deep learning models - MobilenetV2, InceptionV3, ResNet50, and VGG16 and assessed across various supervised learning classifiers. Also, the study used this pretrained models based on transfer learning to predict breast cancer. Across all models, ensemble methods like LightGBM and XGBoost consistently ranked among the top-performing classifiers. These methods demonstrated robust performance, with high accuracy, AUC, and F1-scores. This suggests that ensemble methods are particularly effective at leveraging the features extracted by deep learning models, providing strong classification results. The study also asserts that, choosing the right pretrained model is very important. More complex models like InceptionV3 and ResNet50 provided richer feature sets that led to higher performance. However, this complexity also required more computational resources, making them less suitable for resource-constrained environments. In contrast, MobilenetV2, despite its lightweight design, still performed well, particularly with ensemble methods, making it a desirable choice for scenarios where computational efficiency is a priority. Further fine-tuning of the ML model and incorporating additional features could enhance the system’s performance and broaden its scope.

Transfer learning in deep learning models has the potential to significantly transform breast cancer detection, with profound implications for oncologists, radiologists, and surgeons. By leveraging pre-trained knowledge from large datasets, transfer learning enhances diagnostic accuracy in detecting breast cancer, even with limited specialized data, reducing errors and increasing confidence in mammogram and ultrasound evaluations. This technology alleviates the burden on medical professionals by reducing their dependency on expert annotations, particularly in resource-constrained settings where access to experienced radiologists may be limited. Overall, the integration of transfer learning into breast cancer detection and treatment promises to revolutionize clinical practices, leading to more accurate, efficient, and personalized care for breast cancer patients.

Future works should include; incorporating interpretability tools like Gradient-weighted Class Activation Mapping (Grad-CAM) or Shapley Additive Explanations (SHAP) to visualize image areas influencing model decisions, enhancing clinician insights, using additional datasets (including other imaging modalities) to improve model generalizability and lastly, exploring data augmentation strategies, such as synthetic image generation, to strengthen the robustness of the models.
